# Clinical features associated with cognitive impairment in type I bipolar disorder

**DOI:** 10.1192/j.eurpsy.2026.11190

**Published:** 2026-07-31

**Authors:** N. Smaoui, H. Sghaier, L. Triki, N. Charfi, R. Feki, I. Gassara, S. Omri, M. Maalej, M. Maalej, L. Zouari

**Affiliations:** 1 Psychiatry “C”, Hedi Chaker University Hospital; 2 Department of Functional Explorations, Habib Bourguiba University Hospital, Sfax, Tunisia

## Abstract

**Introduction:**

Cognitive impairment is a common and disabling feature of bipolar disorder type 1 (BD-1).These deficits are influenced not only by the illness itself but also by clinical characteristics such as age at onset, duration of untreated illness, and overall disease course. Identifying the clinical factors associated with cognitive dysfunction is crucial for early intervention and personalized management strategies in BD-1.

**Objectives:**

To explore the links between cognitive deficits and clinical factors associated with bipolar disorder type 1.

**Methods:**

This cross-sectional case-control study was conducted at the psychiatry “C” outpatient unit of CHU Hedi Chaker, Sfax, Tunisia. Fifteen patients with type 1 bipolar disorder (TB-1) and fifteen age- and sex-matched healthy controls were included. Cognitive assessment used the Screen for Cognitive Impairment in Psychiatry (SCIP) scale. Clinical data (age of onset, duration of untreated disease, course) were collected. Statistical tests (Student’s t-test, Chi^2^) compared SCIP scores between clinical subgroups.

**Results:**

The SCIP total score and PST subscore were significantly higher in cases with an age of onset of BD-1 less than 25 years compared with those whose disease started after 25 years (p=0.040; p=0.028).

The SCIP total score was higher in cases with an untreated disease duration of less than 6 months compared with those with an untreated disease duration of more than 6 months, without statistical significance.

SCIP total score was also higher in cases with disease duration of 5 years or less, compared with those with disease duration of more than 5 years, but without statistical significance

**Conclusions:**

These results highlight the importance of evaluating clinical factors in assessing cognitive abilities in TB-1, requiring longitudinal studies to better explore these links.

**Disclosure of Interest:**

None Declared

